# An Alternative Approach to Control Measurements of Crane Rails

**DOI:** 10.3390/s120505906

**Published:** 2012-05-08

**Authors:** Aleš Marjetič, Klemen Kregar, Tomaž Ambrožič, Dušan Kogoj

**Affiliations:** Faculty of Civil and Geodetic Engineering, University of Ljubljana, Jamova 2, 1000 Ljubljana, Slovenia; E-Mails: kkregar@fgg.uni-lj.si (K.K.); tambrozi@fgg.uni-lj.si (T.A.); dkogoj@fgg.uni-lj.si (D.K.)

**Keywords:** control measurements, crane rails, electronic tachymeter, “L” platform

## Abstract

Horizontal and vertical positions of points for the control assessment of crane rails are classically determined separately. The procedure is time consuming and causes non-homogenous accuracy of the horizontal and vertical position of control points. The proposed alternative approach is based on polar measurements using a high accuracy total station instrument and a special platform with two precise surveying prisms fixed on it. Measurements are carried out from a single station thus ensuring a common coordinate system and homogenous accuracy. The position of the characteristic point of a rail is derived from the measured positions of both prisms with known geometry of the platform. The influence of platform non-horizontality is defined, along with its elimination procedure. Accuracy assessment is ensured with redundant measurements. Result of the proposed procedure is a numerical and graphical presentation of characteristic points. The control parameters required in international Eurocode standards are easily determined from them.

## 1. Introduction

In order to ensure safe operation of bridge cranes it is necessary to control the adequacy of crane rails. According to Eurocode 3 standards [1] the span and the elevation difference between rails should be controlled. Classical procedures for control measurements treat the span and the elevation difference separately. The span is controlled using the orthogonal method and the elevation difference using geometric levelling [2]. There are two main disadvantages in the conventional approach. Firstly, two theodolite stations need to be established in the directions of the rails and secondly, horizontal and vertical measurements are carried out separately. In addition, the longitudinal (stationary) position of the control points is not determined. The classical approach is time consuming and does not ensure redundant measurements.

Some alternative methods provided for specific tasks have been published. Kyrinovič and Kopáčik in [3] describe a dynamic measurement method with a special mechanism for the accurate definition of the rail shape. A Leica 360° prism, attached on a special holder, was used. The position accuracy is low due to the dynamic measurements. A method that would assure better position accuracy and precision requires the establishment of a geodetic network [4]. The procedure is expensive, time consuming and the achieved precision is even lower than that obtained using the proposed approach. Advanced methods tend to the complete automation of measurement process. They are based on specially developed equipment and offer real time measurement results [5].

The proposed approach provides simultaneous determination of horizontal and vertical positions of rails from a single instrument station. It is based on the polar surveying method. A precise total station is used along with a special L-shaped platform. Two precise measuring prisms are attached to the platform. We place the platform on the rail at the desired profile points.

The measured polar coordinates are transformed into the Cartesian coordinate system, while the coordinate system is chosen in such a way as to be aligned with the rails. The local Cartesian right-handed coordinate system is used. The obtained coordinates represent a relative relation between the rails.

The proposed approach is better than the classical methods due to its cability for faster measurement of profile points. Higher density of points can be achieved with no effort. Using redundant measurements the precision of the measurements as well as the precision of the results can be assessed. The method ensures homogenous position accuracy of point determination in terms of both horizontal and vertical position. Despite the large number of measurements, the proposed method is still faster and more economical than the conventional approach.

### 2. Instrumentation and Surveying Method

The national standard for crane rails from the Eurocode 3 standards [1] requires the monitoring of:

- The span between rails;
- The elevation difference between rails in each profile.

The actual span can deviate from the projected one by a maximum of 10 mm. The maximum elevation difference must not exceed the value determined by equation. Δ*h_max_= s/600*.

The crane rail is measured using a classical polar surveying method for detail points. Characteristic points are determined indirectly by measuring the position of the “L” platform. According to the required precision and the principles of the method, the proposed approach may be used if two conditions are met:

- A total station providing adequate basic measurement precision of at least 1 mm must be used.
- A target point can be unambiguously signalized in a way that ensures sub millimeter accuracy of centering.

The homogeneity of the measurement precision is maintained by measuring all points from a single instrument station. The instrument must be set in a stable position which enables the visibility of all desired detail points of the rail.

### 2.1. Instrument

According to the required measurement precision derived from standard [1] we define the requirements that the instrument should meet. Given the dimensions of the crane rail, the required accuracy of the point determination can be computed.

Cartesian coordinates of a single detail point are calculated from the measured polar coordinates (*s*—horizontal direction, *z*—zenith angle. *d*—slope distance):

(1) $$\left[\begin{array}{c} x\\ y\\ z \end{array}\right]=\left[\begin{array}{c} \sin s\cdot \sin z\cdot d\\ \cos s\cdot \sin z\cdot d\\ \cos z\cdot d \end{array}\right].$$

Using error propagation law, the precision of coordinate determination can be calculated according to the measurements precisions:

(2) $$\sum_{\text{xyz}}=\text{J}\cdot \sum_{\text{szd}}\cdot {\text{J}}^{\text{T}}.$$

Matrix **J** represents Jacobian and contains derivatives of Equations (1) with respect to each measurement. If **J** is an invertible matrix, the procedure can be inverted. Therefore, the required measurement precisions can be determined according to the desired coordinates precisions:

(3) $$\sum_{\text{szd}}={\text{J}}^{-1}\cdot \sum_{\text{xyz}}\cdot {\left({\text{J}}^{\text{T}}\right)}^{-1}.$$

We are mainly interested in differences between coordinates. The rail span is the difference between the *x* coordinates and the elevation difference equals to the *z* coordinate differences (see Section 3.2) of two points, point 1 and point 2. Coordinate differences between two points of each profile can be written as:

(4) $$\left[\begin{array}{c} \Delta x\\ \Delta y\\ \Delta z \end{array}\right]=\left[\begin{array}{c} x_{2}\\ y_{2}\\ z_{2} \end{array}\right]-\left[\begin{array}{c} x_{1}\\ y_{1}\\ z_{1} \end{array}\right]=\left[\begin{array}{c} \sin s_{2}\cdot \sin z_{2}\cdot d_{2}-\sin s_{1}\cdot \sin z_{1}\cdot d_{1}\\ \cos s_{2}\cdot \sin z_{2}\cdot d_{2}-\cos s_{1}\cdot \sin z_{1}\cdot d_{1}\\ \cos z_{2}\cdot d_{2}-\cos z_{1}\cdot d_{1} \end{array}\right].$$

When we try to use the inversed error propagation law in Equations (4), we face a problem. The Jacobian is neither square nor an invertible matrix. System (4) is therefore expanded with three additional equations for the averages of all three coordinates. Equations complement the system in a way that all the equations are independent and **J** becomes invertible again.

According to the desired coordinate difference precisions, we calculate the required precisions of measurements. They are represented in Table 1.

Simulation results (Table 1) clearly show that in order to achieve millimeter precision of coordinate difference we need the instrument to assure an angular precision of 1″ and distance precision below one millimeter. The dimensions of the treated crane rail are relatively small. In practice crane rails of much larger dimensions are encountered. The required measurement precisions are adequately higher. The proposed approach can therefore be performed only if we can use a total station of the highest precision possible.

### 2.2. Signalization of Detail Points—“L” Platform with Prisms

**Figure 1.**
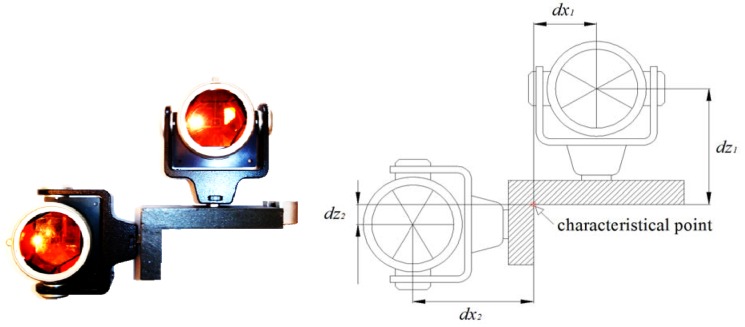
“L” platform for point signalization and its constants.

A special platform (Figure 1) is used for the signalization of detail points. It consists of a holder and two precise prisms force-centered on it. The holder is made of an L-shaped 2 cm thick iron profile. It is 6.5 cm wide and its arms are 7 and 14 cm long. Adapters for precise prisms are attached at both outer surfaces. At the edge a circular level is fixed to the holder to ensure the horizontality of the platform.

The position of the characteristical point, representing the upper inner edge of the rail, can be derived from the measured positions of both precise prisms. Accurate dimensions of the platform need to be known, *i.e.*, offset of both prisms from the edge of the rail.

#### 2.2.1. Calibration of the “L” Platform and Characteristic Point Determination

Each setting of the platform onto a crane rail provides us with two prism center points. We want to represent each setting with one characteristical point. The position of a characteristic point is shown in Figure 1. The centers of measuring prisms are uniquely determinable. The centers of the prisms and the characteristic point lie in the common vertical plane. The coordinate system is defined in such a way that it allows the computation of the characteristic point using the upper prism:

(5) $$\left[\begin{array}{c} x_{\text{KT}1}\\ y_{\text{KT}1}\\ z_{\text{KT}1} \end{array}\right]=\left[\begin{array}{c} x_{1}-dx_{1}\\ y_{1}\\ z_{1}-dz_{1} \end{array}\right]$$

or using the side prism:

(6) $$\left[\begin{array}{c} x_{\text{KT}2}\\ y_{\text{KT}2}\\ z_{\text{KT}2} \end{array}\right]=\left[\begin{array}{c} x_{2}-dx_{2}\\ y_{2}\\ z_{2}-dz_{2} \end{array}\right].$$

Indexes 1 and 2 will hereafter represent the upper (5) and the side (6) prism, respectively. Two computation modes provide us the control and accuracy evaluation of the characteristical point. For such computation parameters, *dx*_1_, *dz*_1_, *dx*_2_, *dz*_2_ defining the geometry of the platform have to be known. Using an indirect approach we determined the values for all four parameters: *dx*_1_ = 58.94 mm, *dz*_1_ = 110.89 mm, *dx*_2_ = 110.52 mm and *dz*_2_ = 15.39 mm. The accuracy of parameter determination is in the order of a tenth of a millimeter. Hereafter they can be assumed to be exact.

#### 2.2.2. Influence of Platform Non-Horizontality

The influence of the non-horizontality of the platform on the determination of prism position was investigated. We propose a computation procedure which minimizes the influence of non-horizontality to the largest possible extent. In order to achieve maximal accuracy, the circular level fixed on the edge of the platform has to be rectified before each use. However, due to limited precision of the circular level, errors of non-horizontality will still appear in the measurements. Effects of non-horizontality should be excluded from the procedure to the greatest extent possible. Rotation of the platform around the given point displaces the upper prism in horizontal direction by:

(7) $$\delta x_{1}=\sin\alpha \cdot \left(dz_{1}+dz_{2}\right)$$

and in vertical direction by:

(8) $$\delta z_{1}=\left(1-\cos\alpha \right)\cdot \left(dz_{1}+dz_{2}\right).$$

The side prism moves contrary in the horizontal direction by:

(9) $$\delta x_{2}=\left(1-\cos\alpha \right)\cdot \left(dx_{1}+dx_{2}\right)$$

and in vertical direction by:

(10) $$\delta z_{2}=\sin\alpha \cdot \left(dx_{1}+dx_{2}\right).$$

Equations (7) to (10) are valid for the rotation around the point defined by the intersection of a vertical line through the upper prism and a horizontal line through the side prism. What is the magnitude of influences caused by non-horizontality on the positions of the prism centres? We assume that horizontality is ensured with the circular level and the accuracy is within the 10′. Considering the geometry of the platform the following values are obtained: *δx*_1_ = 0.3673 mm, *δz*_1_ = 0.0005 mm, *δx*_2_ = 0.0007 mm and *δz*_2_ = 0.4929 mm.

In general the rotation point is not the intersection of lines through prism centers. The actual rotation point is the point where the platform leans on the rail. This point is not defined due to imperfections of the rail shape. However, values of *δz*_1_ and *δx*_2_ are negligible even for greater rotation angles according to the size of the platform. On the other hand, values of *δz*_2_ and *δx*_1_ are noticeable despite the low inclination of the platform and they should not be neglected. To conclude, the vertical component of the position is well defined for the upper prism and the horizontal component is well defined for the side prism. Therefore, to determine the characteristic point Equation (11) should be used instead of Equations (5) and (6):

(11) $$\left[\begin{array}{c} x_{KT}\\ y_{KT}\\ z_{KT} \end{array}\right]=\left[\begin{array}{c} x_{2}-dx_{2}\\ \frac{\left(y_{1}+y_{2}\right)}{2}\\ z_{1}-dz_{1} \end{array}\right].$$

Equations (5) and (6) should serve as control and accuracy assessment.

## 3. Example–Testing of Methods

The proposed method was tested practically in November 2011. The task was to determine the geometry of the crane rail in one of the industrial buildings of the Brestanica thermal power plant (TEB).

To measure the geometry of the crane rails we used a uniform method for determining the positions of the rails in the horizontal and vertical sense. The characteristic points of the crane rail were determined indirectly by measuring the position of the prisms on the “L” platform. We used the classical polar method of surveying, in which we measure the horizontal direction, zenith angle and slope distance to the point. All points were measured from a single station of the instrument. This provides a unique coordinate system for all measured points.

### 3.1. Measurements

We used the Leica Geosystems TS30 total station, with the technical characteristics given in Table 2. Two precise Leica Geosystems GPH1P reflectors were fixed on the “L” platform.

**Figure 2.**
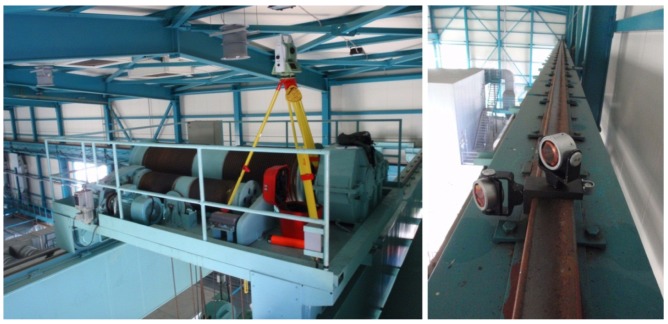
Left: stabilization of instrument; right: stabilization of the “L” platform on the rail.

The instrument was stabilized with a tripod on a stationary crane. The platform was set on the rail every 1.4 m and put in a horizontal position using the circular level. The prisms were pointed towards the instrument. Figure 2 shows the stabilization of the instrument and the measurement point signalization on the rail.

The positions of both prisms were measured twice using the ATR function, which provides homogenous sighting precision for each measuring point on the rail. The platform was placed on each rail 38 times. For each stabilization of the platform each prism was measured twice. As a result we got 304 measured points with the polar coordinates [*s*, *z*, *d*].

### 3.2. Computation of Cartesian Coordinates

**Figure 3.**
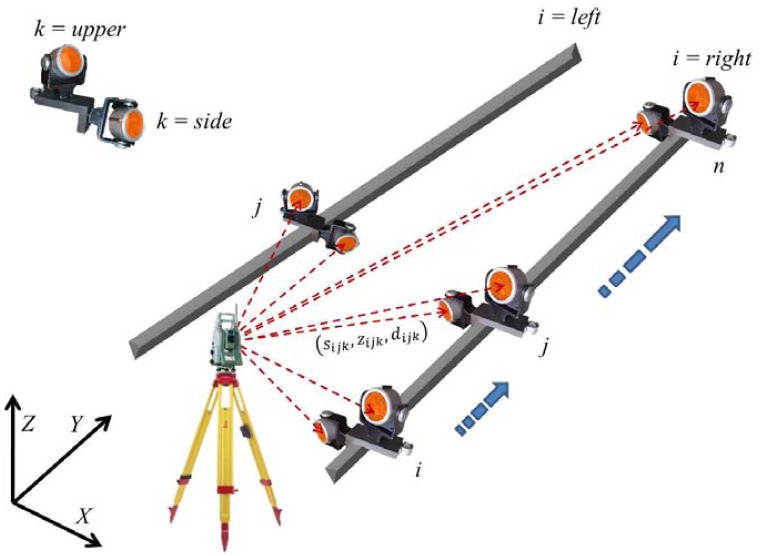
Schematic illustration of measurements.

The results of the polar surveying method (Figure 3) are the horizontal directions *s_ijkl_*, zenith angles *z_ijkl_* and slope distances *d_ijkl_*, where *i* denotes the left or the right rail, *j* a consecutive point on the rail or profile, *k* the upper or side prism on the platform, and *l* the consecutive measurement in each setting of the platform.

For the presentation of the positions of characteristical points on each rail we chose a rectangular Cartesian coordinate system with the following properties:

- the coordinate system unit is the meter,
- the *z* axis is parallel to the gravity axis and pointed up,
- the *y* axis is parallel to the average direction of both rails,
- the *x* axis complements the right-handed coordinate system, therefore it is perpendicular to the average direction of the rails,
- the origin of the coordinate system is arbitrary, but provides the coordinates of all points to be both positive and less than 100.

The computation of point coordinates in the chosen coordinate system can be achieved using the following steps:

- The transformation of points from polar to Cartesian coordinates in the local coordinate system of the instrument can be performed using Equations (1). Since each prism was measured twice, we can immediately check the deviations of repeated measurements and compute the arithmetic mean.
- The *x* and *y* axes of the local coordinate system lie in the horizontal plane, since the instrument was set to a horizontal position. The system has to be rotated around the *z* axis in a way that fulfills conditions iii. and iv. of the desired coordinate system. The rotation of the coordinate system around the *z* axis can be achieved by adding a constant (=orientational direction) to all measured horizontal directions. For each setting of the “L” platform two prisms were measured. Therefore, the rail is represented by two sets (two lines) of points: points belonging to the upper and points belonging to the side prism. The average direction from each of four sets of points was computed by interpolating the line using the least square adjustment. In the adjustment procedure *x_L_* coordinates represent observations and *y_L_* coordinates represent constants. Correction Equations for each set of points are of the form:

(12) ax+b=y.

Azimuth angle for each set is computed from slope coefficient *a* of the line using the following Equation:

(13) $$\nu =\arctan\frac{1}{a}\pm 180{}^{\circ}.$$

The value of orientational direction *o*, used for the rotation of the coordinate system, was computed as the average of all four azimuth angles.

The coordinates of the measured points in the chosen local coordinate system were computed in the same way as in the first step. The only difference was that orientational directions were subtracted from all horizontal directions and that the coordinate system origin was translated 10 meters to the left (the *x* coordinates increased by 10 m) and 5 meters down (the *z* coordinates increased by 5 m):

(14) $$\left[\begin{array}{c} x\\ y\\ z \end{array}\right]=\left[\begin{array}{c} \sin\left(s-o\right)\cdot \sin z\cdot d+10\\ \cos\left(s-o\right)\cdot \sin z\cdot d\\ \cos z\cdot d+5 \end{array}\right].$$

- Twofold measurements of each prism are averaged. Measurement control has already been performed during the computation of the coordinates in the local coordinate system. The results are Cartesian coordinates of all the measured prisms in the desired coordinate system.

### 3.3. Measurement Precision

According to Equation (12) the coordinates of the centres of the two prisms are computed for each position of the platform. Each prism is always measured twice, so we have two positions of each point *t*_1_*=*[*x_t_*_1_*, y_t_*_1_, *z_t_*_1_] and *t*_1_*=*[*x_t_*_2_*, y_t_*_2_, *z_t_*_2_]. The positional difference between them is computed as:

(15) $$\delta t=t_{2}-t_{1}=\left[\delta x,\delta y,\delta z\right].$$

According to differences *δt* for all measured points, we compute parameters, which describe the measurement precision—the average value of differences, δ̄ standard deviation *σ_δt_* and the maximum absolute difference max ‖δ‖The listed values are represented in Table 3.

Given the coordinates of the prism centres and the constants of the platform, the coordinates of the characteristic points for each position of the platform are computed using Equations (5) and (6). The position of the characteristic point for every single measurement is determined in two ways. According to the differences between different solutions the precision of the *x* and *z* coordinates can be analyzed. The precision parameters—the average value of differences *δ̄*, standard deviation *Ơ_δ_* and the maximum absolute difference max |*δ*| are represented in Table 4.

We assume that the differences between the computed values of the coordinates of characteristical point from both prisms on the platform are small enough. The definite values of coordinates are computed from Equation (11), which eliminates the influence of platform non-horizontality.

The precision estimation of the characteristical points follows from the precision of the measured prism centres (Table 4) and from the precision of parameters *dx*_1_, *dx*_2_, *dz*_1_, and *dz*_2_. Based on the error propagation law (propagation of variances and covariances) using Equation (11) the precision of the coordinates of characteristical point can be estimated. The non-horizontality error is ignored:

(16) $$\left[\begin{array}{c} \sigma_{x_{KT}}\\ \sigma_{y_{KT}}\\ \sigma_{z_{KT}} \end{array}\right]=\left[\begin{array}{c} \sqrt{\sigma_{x}^{2}+\sigma_{dx_{2}}^{2}}\\ \sqrt{\frac{1}{2}\sigma_{y}^{2}}\\ \sqrt{\sigma_{z}^{2}+\sigma_{dz_{1}}^{2}} \end{array}\right]=\left[\begin{array}{c} 0.55\text{mm}\\ 0.31\text{mm}\\ 0.51\text{mm} \end{array}\right].$$

It can be noted that the measurement precision is very high. The standard deviations of all three coordinates are far below 1 mm. These values are comparable with the precision of orthogonal measuring method and the method of geometrical levelling.

### 3.4. Determination of Reference Lines and Presentation of Results

All characteristic points of rails were divided into two groups, each representing its own rail. For each rail we have 38 points. Although the position of points (*y* coordinates) of the left and the right rail do not coincide exactly, we assume that the *i*-th points on the left and on the right rail together represent their own profile.

#### 3.4.1. Span of Crane Rails

In the horizontal sense we control the parallelism of the crane rails. In the chosen coordinate system it means a difference of the *x* coordinates of two characteristical points of the same profile. Since we wish to represent the horizontal deviation of each point from the projected position, we must choose characteristic lines that are parallel and spaced for a projected span. In our case, the projected span is 19.300 m. Since we are determining the inner edge of the 0.100 m wide rails, the span between the characteristical points of each profile should be 19.200 m. The reference lines have to be positioned in a way that they represent the average position of all measured points (the average of left and right coordinates) and that they are spaced at a distance of 19.200 m.

A possible way of representing the result of surveying in the horizontal sense is shown in Table 5. The middle column represents the span between the rails and the deviations from characteristical line for left and right rail are in side columns. At the bottom of Table 5 the extreme deviation values of each rail and the extreme deviation values of rail span are given.

**Figure 4.**
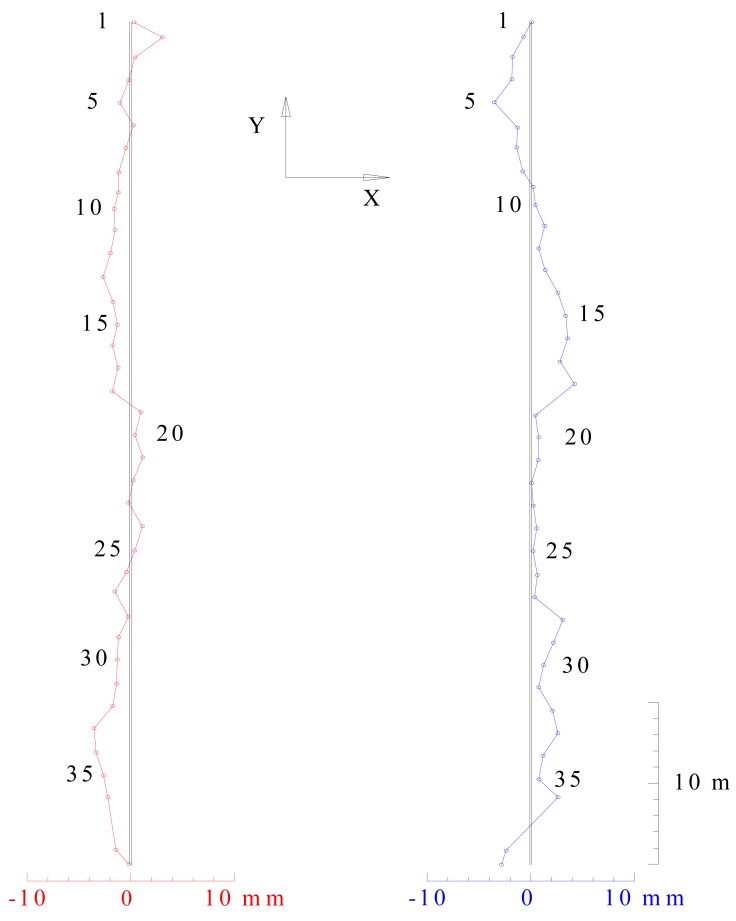
Graphical representation of deviations in horizontal sense (red—left rail, blue—right rail).

It is evident that the deviations are within the tolerances prescribed by the standard (10 mm). Results can be represented graphically as shown in Figure 4.

#### 3.4.2. Elevation Differences of Crane Rails

In the vertical sense it is appropriate to represent the vertical deviation of the rail from its reference horizontal line. The reference horizontal line belongs to an average height of all characteristical points of both rails. In Table 6 the middle column represents the height differences between the left and the right rail in a profile, and the side columns represent the deviations of characteristical points from the average height level. At the bottom of Table 6 the extreme deviation values for each rail and the extreme values for the height differences are given.

**Figure 5.**
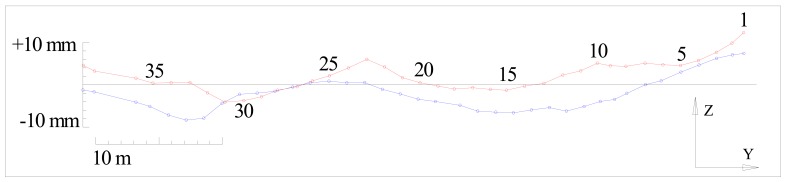
Graphical representation of deviations in vertical sense (red—left rail, blue—right rail).

It is evident that the height differences between the left and the right rail are within the tolerances prescribed by the standard (). (Δ*h*_max_ = 32.2mm) Results can be represented graphically as shown in Figure 5.

## 4. Conclusions

The described method simplifies the process of control measurements of crane rails. It also allows the accuracy assessment from redundant measurements, which was not possible in the conventional way. The method is significantly faster than the classical method. Instead of using two theodolite stations, providing connectivity between both stations, and the use of geometrical levelling, the proposed method allows us to do all measurements from one station with the use of just one instrument (one surveying method). A particular requirement of the method is the use of precise instruments and a calibrated platform with two precise prisms, which allows redundant identification of target points with high precision. The method is based on the simultaneous determination of points in the horizontal and vertical sense that ensures homogeneous precision. The method allows us to obtain data on the exact position of profiles, which ensures detailed modeling of the rail. Knowing the longitudinal position of the characteristical points allows us to perform a Fast Fourier Transformation (FFT) of the data, which provides a deeper insight into the deformation of the rails.

## Figures and Tables

**Table 1.** Calculation of the required measurement precisions for the cases of two profiles (in the beginning and at the end of rail).

*Desired precision of coordinate differences*				
$\sigma_{\Delta x}=\sigma_{\Delta y}=\sigma_{\Delta z}=1\text{mm}$				
*when measuring two points*				
	in the beginning of the rail		at the end of the rail	
	T_left_	T_right_	T_left_	T_right_
horizontal direction	209°	359°	292°	315°
zenith distance	129°	115°	104°	103°
slope distance	7 m	13 m	53 m	55 m
*required precision of measurements*				
*σ_s_*	53.4″	14.6″	3.5″	3.4″
*σ_z_*	62.5″	20.6″	3.4″	3.4″
*σ_d_*	1.8 mm	1.3 mm	0.9 mm	0.9 mm

**Table 2.** Main technical characteristics of the Leica Geosystems TS30 total station.

*Instrument*	
operation interval	−20 ^°^C to +50 ^°^C
resolution of electronic level	2″
*Theodolite*	
minimum distance	1.7 m
standard deviation *σ_iso−THEO_*	0.5″
precision of system ATR *σ_iso−THEO_*	1″ or 1 mm
*Distance meter*	
reference conditions: *n*_0_, *p*_0_, *t*_0_	1.0002863, 1013.25 hPa, 12 °C
range	3.5 km /1 prism, 5.4 km /3 prisms
standard deviation *σ_iso−EDM_*	0.6 mm; 1 ppm

**Table 3.** Statistics of differences between two repeated measurements.

	*x* [mm]	*y* [mm]	*z* [mm]
*δ̄t*	0.033	0.002	0.016
*Ơ_δt_*	0.22	0.17	0.10
max|*δ_t_|*	1.00	0.86	0.49

**Table 4.** Precision parameters of the coordinates of characteristical points.

	*x* [mm]	*z* [mm]
*δ̄*	0.33	0.00
*Ơ_δ_*	0.37	0.55
max|*δ|*	1.00	1.76

**Table 5.** Representation of numerical results in horizontal sense.

*Profile*	*Deviations of the left rail* [mm]	*Span* [m]	*Deviations of the right rail* [mm]
1	0.4	19.2000	0.5
2	3.0	19.1963	–0.7
3	0.7	19.1976	–1.7
4	–0.1	19.1981	–1.9
5	–1.2	19.1978	–3.4
⋮	⋮	⋮	⋮

*max*	3.0	6.3 mm	4.3
*min*	–3.6	–3.7 mm	–3.4

**Table 6.** Representation of numerical results of surveying in vertical direction.

*Profile*	*Deviation of left rail*[mm]	*Height difference*[mm]	*Deviation of right rail* [mm]
1	12.7	5.0	7.7
2	10.0	3.0	7.0
3	7.5	1.3	6.2
4	5.6	1.2	4.4
5	4.8	1.9	2.9
⋮	⋮	⋮	⋮

*min*	12.7	9.0	7.7
*max*	–4.5	–1.7	–0.8
